# “It’s already in your body and it’s preventing”: a qualitative study of African female adolescent’s acceptability and preferences for proxy HIV prevention methods in Cape Town, South Africa

**DOI:** 10.1186/s12889-023-16955-3

**Published:** 2023-11-02

**Authors:** Lauren Fynn, Katherine Gill, Melissa Wallace, Millicent Atujuna, Menna Duyver, Penelope Ngcobo, Hans Spiegel, Alex Rinehart, Sybil Hosek, Linda-Gail Bekker

**Affiliations:** 1 Desmond Tutu HIV Centre, University of Cape Town, Cape Town, South Africa; 2 Department of Health and Human Services, Kelly Government Solutions, Contractor to National Institute of Allergy and Infectious Diseases, National Institutes of Health, Rockville, MD USA; 3 ViiV Healthcare, Research Triangle Park, NC USA; 4 Stroger Hospital of Cook County, Chicago, IL USA

**Keywords:** South Africa, HIV prevention, Contraception, Adherence, Preference, Adolescent girls

## Abstract

**Background:**

Advances in biomedical HIV prevention will soon offer young women a choice of HIV prevention methods, including various pre-exposure prophylaxis (PrEP) modalities such as daily oral pills, dapivirine vaginal ring, and long-acting injectable agents. By understanding preferences for contraceptive methods, we may draw analogies for the HIV prevention needs of young women.

The UChoose Study was an open-label randomised cross-over study designed to evaluate the acceptability and preference for several contraceptive options as a proxy for HIV prevention methods that use similar types of administration. The study enrolled healthy HIV uninfected young women aged 15 to 19 years. At enrolment, participants were randomly assigned to a contraceptive method for a period of 16 weeks in the form of monthly Nuvaring® (vaginal ring), daily combined oral contraceptive (daily pills), or bi-monthly injectable contraceptive (injectable). After 16 weeks, participants crossed over to another contraceptive method, and those who had received the injectable and the daily pills received the vaginal ring for another 16 weeks, whereas those who had received the vaginal ring were able to choose between the injectable and daily pills, to ensure that all participants tried the vaginal ring—the least familiar option to the study population.

**Results:**

Thirty-three participants were purposively recruited to participate in seven focus group discussions (FGD) and completed a pre-survey for their assigned group. Our sample comprised 14 participants randomised to use of the vaginal ring and daily pills and 19 participants randomised to use of the vaginal ring and injectable. For most participants, their preferences for a prevention method were based primarily on their desire to avoid negative aspects of one method rather than their positive user experience with another method. Most participants expressed initial hesitancy for trying new contraception method products; however, a lack of familiarity was moderated by a strong interest in diverse user-controlled prevention methods. Participants valued methods that had infrequent dosing and simplified use requirements. The injection and vaginal ring were preferred over daily pills as a potential HIV prevention method.

**Conclusion:**

Expanding the availability of diverse products could provide adolescents with multiple choices in HIV prevention for the uninitiated.

**Trial registration:**

ClinicalTrials.gov (NCT02404038). Registered March 31, 2015—Registered.

**Supplementary Information:**

The online version contains supplementary material available at 10.1186/s12889-023-16955-3.

## Background

Significant investments to combat the HIV epidemic in sub-Saharan Africa (SSA), have resulted in progress towards new HIV-1 prevention options; however, the epidemic continues to disproportionally affect adolescent girls and young women (AGYW) [1, 2]. Approximately 4900 young women between the ages of 15 and 24 contract HIV every week [3]. Six out of every seven new HIV infections among adolescents in sub-Saharan Africa between the ages of 15 and 19 are among AGYW. The prevalence of HIV in girls and young women (15–24) is double that of young males. AGYW made up 63% of all new HIV infections in SSA in 2021 [3]. In South Africa, adolescent girls and young women account for over two-thirds of new HIV infections and acquire HIV at twice the rate of their male counterparts [1, 3, 4]. Over the past 30 years, UNICEF has recognized the importance of HIV prevention within this risk group and set goals to reduce HIV incidence by increasing the availability of HIV prevention options [4–7].

Advances in biomedical HIV prevention will soon offer young women a choice of multiple female-controlled HIV prevention methods, including oral antiretroviral pre-exposure prophylaxis (PrEP), the dapivirine vaginal ring, and long-acting injectable cabotegravir [8–11]. Clinical trials have shown that new prevention methods such as oral PrEP, the dapivirine vaginal ring, and long-acting injectables have the potential to confer protection against HIV [12–15]. However, product choice and acceptability have multiple dimensions based on individual and contextual factors and product features [16–19]. As with contraceptive choices, HIV prevention options adopted by adolescent girls and young women will likely depend on which methods they find most advantageous and acceptable [20–23]. Research has also highlighted potential user concerns surrounding side-effects, availability, efficacy, and cost of HIV prevention options [14, 15, 24–26].

The UChoose Study was designed to evaluate the acceptability and preference for several contraceptive options as a proxy for HIV prevention methods with similar routes of administration [27]. The open-label randomised cross-over study occurred over 32 weeks among healthy, HIV-uninfected, female adolescents aged 15 to 19 years. At enrolment, participants were randomly assigned to a contraceptive method for a period of 16 weeks in the form of monthly Nuvaring® (vaginal ring), daily combined oral contraceptive (daily pills), or bi-monthly injectable contraceptive (injectable). After 16 weeks, participants crossed over to another contraceptive method, and those who had received the injectable and the daily pills received the vaginal ring for another 16 weeks, whereas those who had received the vaginal ring were able to choose between the injectable and daily pills, to ensure that all participants tried the vaginal ring—the least familiar option to the study population. The use of proxy options provided the opportunity for participants to develop an understanding of product features and capture user experiences and preferences, enabling extrapolation to possible modes of delivery for current and future PrEP products for AGYW. From the main study, participants consistently reported a preference for the injectable method over other forms of HIV prevention, followed by the ring and finally, the pill [27].

By understanding preferences for contraceptive methods, we may draw analogies for the HIV prevention needs of young women [27]. While a number of studies have explored women’s experiences of using different modalities for PrEP delivery, there has been less focus on adolescents younger than 18 years. The needs and preferences of adolescents may differ from those of older women due to the distinct developmental and behavioural differences between adult women and female adolescents. The work presented here focuses on comparative feedback from participants who had used the vaginal ring and one other contraceptive method and were recruited to participate in a focus group discussion to explore factors related to acceptability and preferences for each method. In addition, while interested in the participants’ experiences with each method, we further engaged participants to extrapolate those experiences to imagined experiences with HIV preventive strategies.

## Methods

### UChoose study

The UChoose study was an open-label, randomised cross-over trial conducted between September 2015 and July 2017 [27]. The UChoose trial design and study results are reported elsewhere [27], but briefly, the trial design evaluated the acceptability and preference for contraceptive options as a proxy for similar HIV prevention methods. The study enrolled 130 healthy, sexually active, HIV-negative female adolescents, aged 15 to 19 years within a peri-urban, low-income community in Cape Town. Participants were willing to be randomised to two contraceptive methods after written informed consent was obtained from participants over 18 years. At study completion, 116 participants had used the vaginal ring, 73 injections and 48 daily pills. Within the main study, 94/130 (74.6%) participants completed all follow-up visits including the cross-over visit and thus actively used two products over the course of their study participation. Participants younger than 18 years, provided assent, while written consent was sought from parents/legal guardians in the preferred language. Additional consent was requested for participants identified for participation in the Focus Group Discussions. Participants were ineligible if they had medical contraindications to study products, were living with HIV, or if they were pregnant or had the intention to become pregnant in the next eight months and/or if they were unwilling to be randomised to a contraceptive method.

At enrolment, participants were randomised to a contraceptive method for a period of 16 weeks in the form of a monthly vaginal ring (*n* = 45), a daily combined oral contraceptive (daily pills, *n* = 45), or bi-monthly injectable contraceptive (injectable, *n* = 40). Participants “crossed over” to receive a different contraceptive for an additional 16 weeks, and those who had received the injectable and the daily pills received the vaginal ring for another 16 weeks, whereas those who had received the vaginal ring were able to choose between the injectable and daily pills, to ensure that all participants tried the vaginal ring. At each scheduled visit, participants were provided contraceptive education, HIV testing, and risk reduction counselling, as well as testing and treatment for sexually transmitted infections (STI) as needed. In addition, participants were requested to complete interviewer assisted behavioural questionnaires and clinical outcome tests.

The UChoose study protocol, including its qualitative component, was approved by the University of Cape Town (UCT) Health Science Research Ethics Committee (720/2014), the US National Institutes of Health, NIAID Division of AIDS, and registered in the public registry database of ClinicalTrials.gov (NCT02404038).

### Data collection and analysis

This analysis includes data from the UChoose qualitative procedures conducted in April 2018, approximately one month after the final participant study exit. This qualitative study involved the participants in hypothetical situations with identical HIV prevention modalities in addition to exploring acceptability, adherence, and decision variables for contraceptive methods. In our context, participants were purposely recruited at the endpoint to ensure that participants could speak to their experience of using two different contraceptive methods. Study field staff followed up with these participants to confirm their willingness to be selected for FDGs and excluded who may have relocated or did not have active contact information. The emphasis for staff was to invite participants based on their use of at least two products and completion of all study visit activities, not necessarily adherence to two products. Participants included in the final FDGs had all used the vaginal ring and at least one other product over their study participation. The sample sizes for these qualitative activities were designed to provide enough breadth and diversity of answers from each Group category while also assuring data saturation. A semi-structured FGD guide was developed based on a comprehensive literature review guided by the study aims and objectives.

The FGD included two activities; completion of an interviewer assisted modified ORTHO pre-survey, described below followed by the participant driven FGD. This activity provided both the participants and interviewers with a way to frame their conversation and ensure debate on differing opinions. The pre-focus group survey is the modification of the ORTHO birth control assessment tool (Table 1) which was used to allow participants to frame some of their initial feedback focused on the research subject exploration of participant preferences for hypothetical HIV prevention modalities. The modified ORTHO birth control assessment tool was available to review as reference material throughout the FGD to facilitate participants’ understanding and exploration of their preferences for hypothetical HIV prevention methods. The paper-based modified ORTHO pre-survey assessed the factors that contribute to overall contraceptive acceptability and user satisfaction. Utilizing the eight domains described within the ORTHO BC by Colwell et al. [10], participants were asked to not limit their discussion to only their experience of use but also describe potential concerns as they envisioned future potential HIV prevention methods (Table 1).

**Table 1:** The modification of the ORTHO birth control assessment tool

Ortho BC-sat	Modified Ortho BC-Sat
Pregnancy Prevention	HIV Prevention modality
Ease of Use/Convenience	Ease of Use/Convenience
Compliance	Adherence
Lifestyle Impact	Lifestyle impact
Symptom/Side Effect, Bother	Symptom/Side Effect, Bother
Menstrual Impact	Sexual impact
Future Fertility Concerns	Future HIV infection Concerns
Assurance/Confidence	Assurance/Confidence
Overall Satisfaction	Overall Satisfaction

The semi-structured FGD schedule comprised a series of thoughtfully designed questions that aimed to explore participants’ preferences and experiences related to the HIV prevention method. The schedule covered various topics, including (1) experience with each contraceptive method, (2) preferred contraceptive methods, and (3) preference for biomedical and behavioural HIV prevention methods discussed. To initiate the discussions, participants were asked open-ended questions to gauge their initial impressions and preferences regarding different prevention methods used such as “Can you tell me about the contraception method you are using or have used in the past?”. Subsequently, the FGD facilitators utilized follow-up questions to delve deeper into participants’ underlying motivations, concerns, and experiences related to each of the methods. These follow-up questions sought to uncover the factors influencing participants’ preferences, such as efficacy, convenience, side effects, and personal beliefs. By employing a structured approach, the FGD schedule aimed to elicit rich and detailed responses, offering valuable insights into the participants’ perspectives on the proposed HIV prevention method. This included questions such as “Is there another HIV prevention method that you would prefer more? Why/why not?” Participants were asked about their experiences and opinions based on their use of contraception methods with the intention of having a safe, honest, and non-judgmental open dialogue.

The FGDs each included time to complete informed consent followed by the pre- survey, time for the local staff member to provide all participants with reference materials and encouraging participants to answer each of the questions to the best of their ability. FGDs were held in a pre-arranged quiet and discrete location and lasted up to 60 min. All FGD participants were offered refreshments and received reimbursements. The FGDs were co-facilitated by trained local study staff and socio-behavioural leads in local languages. All FGDs were audio-recorded, transcribed verbatim, and translated into English.

Descriptive statistics were used to present demographic, background data, and survey data using SPSS, version 25.0 [28]. We used framework analysis [29] to develop a qualitative codebook to organize the data according to key themes with a focus on acceptability and preferences (Guide I). A framework analysis is a systematic structure for managing, analysing, and detecting themes, and it works especially well with large quantities of text [29]. Establishing a thematic framework relevant to the research subject is central to the technique. This enabled the analysis team to label, categorize, and arrange data per core subjects, concepts, and categories [29]. Qualitative coding was conducted using NVivo 11 software [30, 31]. Two coders independently coded all FGD transcripts, and then transcripts were assessed for inter-rater reliability. Any discrepancies in coding were discussed and resolved in discussion with senior researchers as per the framework analysis process. Summaries of textual excerpts for each code were analysed for salient themes related to acceptability and preferences of the vaginal ring versus the injectable and daily pills, and contextual factors informing stated product preferences of three hypothetical antiretroviral-based HIV prevention modalities. To help participants, especially adolescents, better understand and reflect on the hypothetical use of HIV prevention products, trained local study staff provided reference materials at the beginning of each group. These materials included sample products and detailed diagrams, which illustrated how the products could be used. This approach aimed to elicit more thoughtful and accurate responses from the participants when discussing their hypothetical use for HIV prevention.

## Results

A total of 48 of 94 participants were invited to attend the FDGs with a target of 5–6 participants per group. Participants were allocated to either Group A: vaginal ring and daily pills or Group B: vaginal ring and injectable FDGs. This resulted in 3 FGDs from Group A and 4 FGDs from Group B (Table 3). The facilitators began the session once a minimum of 3 participants were present. Lack of participation in the study was due to absenteeism on the day of the focus groups resulting in the attendance of 33 participants. Thirty-three UChoose participants were included in this qualitative analysis. Table 2 describes the participants’ demographic characteristics and sexual behaviour at enrolment as well as contraceptive method assignment throughout the study. At the time of enrolment, the majority of the participants lived with their parents, attended school, and had completed up to grade 9. The average age of sexual debut was 15, with the majority having one main sexual partner in the past year and using condoms half the time to always during their last sexual act. There were no notable differences across the groups’ demographics.

**Table 2:** Demographics, reported sexual behaviour, pregnancy history at enrolment of adolescent women in the UChoose study

Demographic factor	Overall (*n* = 33)
Age (Years)	17 (16 to 18)
Living with parents (%)	27 (82%)
Use of alcohol in preceding 12 months [n/N (%)]	4 (12%)
Education [n/N (%)]	
School attendance	29 (88%)
Highest grade completed	9 (27%)
Out of school	4 (12%)
Sexual behaviour	
Age of sexual debut (Years)	15 (14–18)
Number main sexual partners past year	1 (1 to 3)
Multiple sexual partners past year [n/N (%)]	29 (88%)
Partner had multiple sexual partners past year [n/N (%)]	2 (6%)
Condom use at last sexual act [n/N (%)]	28 Half time – Always (85%)
Intergenerational (≥ 5 years age difference) sex [n/N (%)]	2 (6%)
Transactional sex [n]	0
Anal sex [n]	0
Felt she was at high risk (≥ 70%) of acquiring HIV [n(%)]	1 (3%)
Felt she had high level of protection (≥ 70%) against acquiring HIV [n(%)]	18 (55%)
Contraceptive use [n (%)]	
Vaginal Nuvaring and Daily Pill	14 (42%)
Vaginal Nuvaring and Injection	19 (58%)

Using survey questionnaires, we calculated each variables values at baseline

Data is shown as number, Years [median (IQR)], otherwise indicated as Number (%)

All participants agreed to complete the modified ORTHO pre-survey prior to the FGDs. Our sample was comprised of Group A—14 participants randomised to use vaginal ring and daily pills, and Group B—19 participants randomised to use vaginal ring and injectable (Table 3). We collated codes from the analysis of FGD transcripts into three broader emerging themes: (1) acceptability as related to experiences of product use, (2) preferred contraceptive products, and (3) preference for biomedical and behavioural HIV prevention methods.

**Table 3:** Focus group representation per arm

	***Arm (Group A or Group B)***	***Attendants***
***Session 1**** (Target 5–6)*	Group A: vaginal ring and daily pills	5
***Session 2**** (Target 5–6)*	Group A: vaginal ring and daily pills	4
***Session 3**** (Target 5–6)*	Group B: vaginal ring and injectable	5
***Session 4**** (Target 5–6)*	Group B: vaginal ring and injectable	5
***Session 5**** (Target 5–6)*	Group A: vaginal ring and daily pills	5
***Session 7**** (Target 5–6)*	Group B: vaginal ring and injectable	4
***Session 8**** (Target 5–6)*	Group B: vaginal ring and injectable	5

Please note that session 6 was marked as incomplete as we did not have enough participants arrive for the session. The facilitators began the session once a minimum of 3 participants were present

### Quantitative findings

**Fig. 1:**
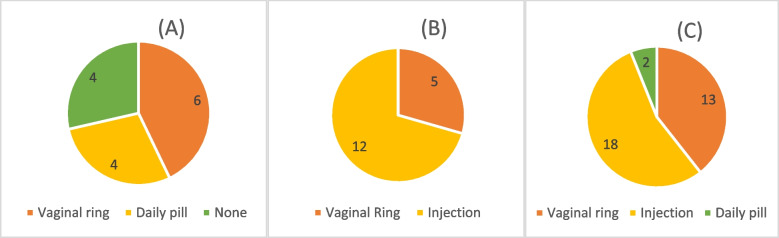
HIV Prevention product preference of adolescent women in the UChoose study. **A** 42% (*n* = 6/14) preferred the vaginal ring; 29% (*n* = 4/14) preferred the pill; 29% (*n* = 4/14) an alternative to these two options or not to use anything at all. **B** 70% (*n* = 12/17) preferred injectables; 30% (*n* = 5/17) preferred the ring. **C** Overall preference scoring—55% (*n* = 18/33) preferred injectable option, followed by 39% vaginal ring (*n* = 13/33) and 6% daily pill (*n* = 2/33)

The figure, labelled Fig. 1, presents data from the pre-focus group survey and reflects a summary of participant preferences and choices regarding contraceptive and HIV prevention methods. 42% (*n* = 6/14) of participants in Group A said they preferred the vaginal ring over the use of a daily pill, while 29% (*n* = 4/14) participants indicated that they preferred the pill over the ring (Fig. 1A). In addition, 29% (*n* = 4/14) of participants said they would prefer an alternative to these two options or not to use anything at all. In contrast, participants who compared the vaginal ring to the injectable (Group B) were more willing to continue using injectables (*n* = 12/17, 70%) while 30% (*n* = 5/17) preferred the ring (Fig. 1B).

**Fig. 2:**
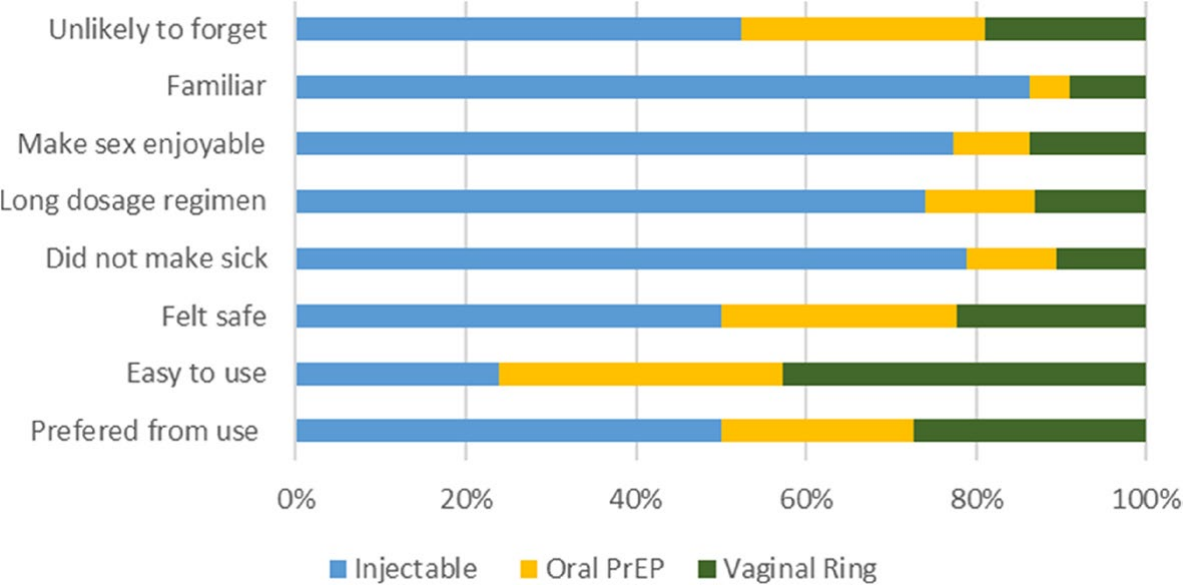
Reasons for preference of adolescent women in the UChoose study

When asked about preference for potential HIV prevention methods, the majority of participants chose an injectable option, (*n* = 18/33, 55%), followed by vaginal ring (*n* = 13/33, 39%) and daily pill (*n* = 2/33, 6%) (Fig. 1C). Overall, participants most frequently mentioned ease of use, method familiarity, and minimal privacy and adherence challenges (Fig. 2).

### Qualitative findings

We present qualitative themes categorized into three areas: (1) acceptability as related to experiences of product use, (2) preferred contraceptive products, and (3) preference for biomedical and behavioural HIV prevention methods.

### Acceptability for contraceptive prevention products

#### Desire to know about various contraceptive options vs. concern about using unfamiliar products

Participants were asked to think back to their initial thoughts about the study products as well as acceptability at the study product cross-over. Users who were contraceptive naïve reported some initial apprehension for each contraception product; however, overall, participants reported that they found all methods acceptable at study enrolment. Most described how acceptability was initially affected by their interest in having a ‘healthy life’ and their willingness to try novel contraception products.

*“I was using injection as a family planning method, so I was interested in joining the study because I wanted to try other family planning methods.”* (Participant Group B - vaginal ring and injectable)

*“I also enjoyed being in the study because it also introduced us to some of the things like how we should… like other options of prevention other than the injection that most of us know, it introduced us to the pill and the ring that I did not know of.”* (Participant Group A - vaginal ring and daily pills)

#### Factors affecting acceptability over time for various contraceptive options

Participants reported how acceptability may have varied for them over time, particularly at the study product cross-over. Of note, several of the factors that may have influenced the acceptability of prevention methods included reservations about product efficacy, duration of protection, and dosage frequency. As described by one participant:

*“Because they do not feel comfortable with the ring and then with pills, they forget most of the time, but with injection, they cannot easily forget it, once it is injected in you it is there.”* (Participant Group B - vaginal ring and injectable)

### Preferences for contraception prevention products

#### Physical comfort with having something in the vagina vs adherence challenges of pills

Differences in preference were explored with participants by discussing future choices and how the modified Ortho BC-sat domains (Table 1) would affect their choice of the contraceptive method given their experience of using two contraception products. For most participants, their preferences for a contraception method were based purely on the trade-off of negative attributes for each method rather than focusing on positive user experiences. Specifically, participants appreciated products that offered low participant administration and maintenance:

*“Nuvo ring because I was comfortable using it and I liked it very much. I was using pills, and I did not like them because I forgot them.”* (Group A - vaginal ring and daily pills)

As participants discussed their preference for products that were easy to use, they also discussed ways in which their adherence was affected by having to control for when, and possibly where, some products were used. For some, this meant consciously choosing to take the product or subconsciously forgetting to use the product.

*“I don’t like the pills. It’s just like that; I have to take it every time, the pills so if I forget it, I can be pregnant.”* (Group A - vaginal ring and daily pills)

#### Adherence challenges of pills: frequency of having to attend to the product

As suggested by participants, acceptability considerations made them also think of the way in which these products are used over time thus a few made dosage recommendations. A few participants from Group A volunteered suggestions for alternative dosage possibilities for future products. Participants expressed an interest in rings that only needed to be replaced bi-monthly and tablets that only needed to be taken weekly.

*“It is the pills because I used to forget them every day; sometimes, I would bring them back here. If we can change pill dosage like taking it once a week.”* (Group A - vaginal ring and daily pills)

#### Adherence challenges of pills: conforming to their way of life

These adherences challenges were closely related to the ways which product use would impact their lifestyle. Participants described situations which they believe demonstrated how the prevention products should “fit in” with their lifestyles, allowing for control over one’s reproductive health but also the choice to maintain privacy regarding their sexual activity.

*“And then there’s partying, sometimes you get there early and there is a vibe, and you don’t want to miss out. You forget the pills now and then you have to start from the beginning.”* (Group A - vaginal ring and daily pills)

#### Trade-offs of vaginal ring vs participants concerns of injections

Aside from these barriers, participants expressed concerns about the injectable contraception side effects. Within these FGDs, participants reported experiencing low or no side effects while on the daily pills or vaginal ring. However, the participants who used the injection, commonly framed side effects negatively while describing pain at the injection site, abnormal uterine bleeding, and/or perceived physical weight changes.

*“For me, the ring. The injection is just tricky as it stands because its side effects never end. They are always there; it is either you lose weight, or you gain weight, or your leg becomes numb every time, and it is a problem.”* (Group B - vaginal ring and injectable)

*“I prefer the ring. Because sometimes when you’ve injected yourself with the Nuristerate you don’t see your period, you don’t go to period, but when sometimes you use the ring you can see your periods, even if it’s just a drop.”* (Group B - vaginal ring and injectable)

#### Desire for a covert method across products

In the FGDs, participants expressed concerns about the social perceptions of contraceptive products and the potential for others, particularly parents and sexual partners, to find out. Some participants preferred covert methods, such as the injectable, due to the ability to hide use from parents. Others highlighted the importance of being open with sexual partners about using the vaginal ring to avoid misunderstandings. The choice for covert use ultimately depended on individual participant preferences.

*“I think if you didn’t tell your partner when you were using the ring, then maybe when you have sex the ring might come out, and then what’s she gonna say? Then your boyfriend is gonna think otherwise of you, but its better if you tell him that ‘I am in the study and I am using this method’ then everything is fine.”* (Group B - vaginal ring and injectable)

*“Maybe you hide your pills because you don’t want your parents to see them, so the injectable is good.”* (Group A - vaginal ring and daily pills)

In contrast, one participant explained how social opinion, not just her partner or parents, has affected her personal beliefs to some extent. For this participant, pill taking is considered a behaviour of disease associated with HIV treatment rather than prevention.

*“[I prefer] the ring. My mind is like you are sick if you are taking a pill, so it does not work for me.”* (Group A - vaginal ring and daily pills)

#### Thinking of future choices for adolescents

Participants were encouraged to verbalize any feedback which we could learn from as we plan for future products. For example, some participants referred back to the beginning of the study and spoke about how they were concerned about the efficacy of each product while acknowledging that adolescents often struggle to use products as instructed.

*“I prefer injection because I am using it already, but I am skeptical about other things regarding the ring, but it [the injection] works. There is nothing wrong with it.”* (Group B - vaginal ring and injectable)

Finally, participants were provided a period of reflection in which they were encouraged to consider all the feedback from the session and information they had learned about all three products throughout the study. Participants were then asked to confirm their preference for, or justify a change from, their contraceptive choice indicated at the beginning of the session. As shared by a participant:

*“I would say the injectable because at least… especially people who forget or who drink a lot, because you can stay the whole month not going to the clinic, but the injectable is already in your body and it’s preventing.”* (Group B - vaginal ring vs injectable)

### Preferences for potential HIV prevention products

During the FDGs, the majority of participants selected the injectable option as their preferred potential HIV prevention method, followed by the vaginal ring, and then the pill. Participants emphasized the importance of ease of use and favoured long-acting formulations. Participants were asked to substantiate their HIV prevention option with consideration to available HIV prevention options such as condoms and non-penetrative sex.

*“I choose the injection first because I trust it and when I get it, I do not have side effects. Also, choose condoms because it prevents me from getting STIs”* (Group B - vaginal ring vs Injection)

Overall, more than half of the participants chose the injection while emphasizing the importance of minimal side effects and additional protection practices that must be considered. As highlighted by participants who describe the injectable and vaginal ring options as ‘*I choose this because it is easy to use and are not forgettable*’ and *‘because they are easier things I can do’*. Moreover, ‘[I] *cannot even forget them’*. Nevertheless, while the injection was described as irrevocable and thus most preferred, there exists enough variation to indicate that a range of methods need to be offered to meet the needs and preferences of all AGYW.

*“You can take the pills and sometimes you forget, but with the injection, it’s only once and then you would come back.”* (Group A - vaginal ring and daily pills)

## Discussion

This paper presents some of the first quantitative and qualitative data exploring the acceptability and preference of adolescent girls and young women in the UChoose study for contraceptive options as a proxy for HIV prevention methods. Our results highlight three key findings: first, the burden of daily use and patterns of poor execution were the most consistently described concerns for future acceptability and preferences for HIV prevention products. Second, despite the initial hesitancy that adolescents aged 15–19 may experience with new products, there is a strong interest in diverse user-controlled prevention methods. Third, participants described adherence barriers, underscoring the need for products that are easy to use and integrate well into adolescents’ lives. Fourth, the ease of administration and long-lasting efficacy were primary factors linked to acceptability and preference among South African adolescents over time. Indeed, the most commonly preferred methods were an injectable option or a vaginal ring from the three pregnancy prevention options. Overall findings revealed adolescents were enthusiastic about HIV prevention that may improve their health. The overall preference feedback for the injectable HIV prevention method was indeed consistent with the main study [27]. Participants consistently reported a preference for the injectable method over other forms of HIV prevention. This provides important confirmation of the initial findings and strengthens the validity of the results.

Similar to acceptability and preference data from other at-risk groups, adolescent women in our study described the relative trade-offs of each method [8, 9, 11]. Participants described how future interest in these products would largely be dependent on how individuals use the products in their daily lives [12]. Although oral pills and monthly injections were commonly known methods, participants were open about utilizing new methods. Although participants had low initial awareness of vaginal ring options, their interest in a vaginal ring option highlights the critical role of demand creation and educational outreach in the success of new HIV prevention methods, especially among a product-naive population [13–15]. These results are consistent with other studies of product preference conducted among older at-risk women [8, 9, 21].

Similar to evidence found in other HIV prevention literature, we found that participants commonly reported these concerns for both the vaginal ring and daily pills method, demonstrating a lack of confidence in their ability or agency to use these products habitually [20, 22, 23]. As one example of strategy for this age group, participants frequently recommended increasing the pill's dosage length to one tablet every two or four weeks. Establishing belief in product efficacy within the first months of use was a key lesson. Future research might address ways to reduce declining personal confidence, self-judgment for incorrect use, and eventually poor adherence [14, 32].

While participants were asked to extrapolate from their contraceptive experience, participants emphasized adherence issues more than how to solve them. Participants acknowledged that no single product had all the favourable characteristics but voted for prevention that ensured ease of use, had ‘full’ protection, and infrequent dosing [23, 33]. While other studies have reported side effects as one primary factor affecting the choice of HIV prevention and/or contraception dosage [34, 35], in this sample only a minority of participants expressed concerns about side effects. Rather, side effects of HIV prevention methods were discussed as abstract concepts with participants assuming that there would be some negligible or no side effects.

Consistent with previous research on the acceptability of new HIV prevention options [36–38], the majority of participants predicted that other adolescents would prefer the injection because of its discretion; however, client-focused counselling and social support remain a recommendation for this age group, as demonstrated in similar trial communities [38–40]. The timing of disclosure and the depth of information provided to participants may have influenced communication between the participants and others in their social network about contraceptives and related HIV prevention methods [33, 41]. Multiple factors, such as community attitudes and parental, peer, and partner support, were found to reinforce existing risk behaviours or drive safer choices.

We acknowledge that participants may still have given socially desirable answers, despite FGDs having been conducted in an open and non-judgemental manner. Of similar importance, a small study in a single district may influence generalizability to other adolescent populations in the country. Additionally, the FGDs were conducted in 2018 following study completion and relied heavily on retrospective self-report data that is subject to recall bias and misreporting. In light of the study design, FGD participants were selected at the end of the study from the sample of participants who had used at least two of the prevention methods as we required participants to describe and make contrasting comparisons regarding their experience of use. In this study, we attempted to mitigate any recall bias by providing participants reference material at the beginning of each group including the sample products and detailed diagrams and reemphasizing their key role in giving candid feedback [29, 42]. In our context, participants were purposely recruited at the endpoint to ensure that participants could speak to their experience of using two different contraceptive methods; however, to minimize biases further, we suggest serial qualitative data collection data collection in future iterations of similar study*.* Additionally, adherence was not a criterion for selection in this study because the study's focus was on participants’ preferences and experiences with different contraceptive methods and HIV prevention methods, rather than their actual use of these methods. This approach can provide a more comprehensive understanding of participants’ preferences and experiences with the different methods, which can be useful in designing more effective and acceptable interventions in the future*.* Finally, within the UChoose trial, participants were allowed to choose between the injectable and daily pills after the cross-over. This may have introduced a bias towards the more acceptable or preferred method when compared to the vaginal ring. Throughout participants’ narratives, the complexity of the acceptability and their experiences of adherence, or nonadherence and to a lesser extent privacy concerns, complement UChoose trial results [27].

## Conclusions

As evidenced through HIV prevention trials among at-risk populations, adolescents appreciated simplified use and infrequent dosing, minimal or non-existent side effects, assurance of product efficacy, and methods that alleviated worries around forgetting doses. Our study underscores the significance of user-centric options in enhancing long-term adoption and trustworthiness, thus contributing valuable insights for the future development and promotion of HIV prevention products. We also recommend that the social support from appropriate staff will be essential for the uptake and use of new product use. HIV prevention option for women requires leveraging existing social support groups and emphasizing multiple method options for young women. Although injections were most preferred, expanding the availability of daily pills and the vaginal ring can provide adolescents with multiple choices in HIV prevention technologies, understanding that prevention option naïve users need varying options. In conclusion, this study’s findings provide further insights into factors that are key to young women’s preferences of HIV prevention methods. As enthusiasm grows for the development of products, product developers and providers should also be aware of this knowledge to empower young women with tools to better protect their health globally.

### Supplementary Information

**Additional file 1. **

## Data Availability

De-identified data presented in this manuscript will be shared upon reasonable request and receipt of a completed data request form.

## Abbreviations

UCT: University of Cape Town
PrEP: Pre-Exposure Prophylaxis
FGD: Focus Group Discussions
AGYW: Adolescent Girls and Young Women
Vaginal Ring: Nuvaring®
Daily Pills: Daily Combined Oral Contraceptive
Injectable: Bi-Monthly Injectable Contraceptive
STI: Sexually Transmitted Infections
SSA: Sub-Saharan Africa
HIV: Human Immunodeficiency Virus
