# Supplementary material for: “It’s already in your body and it’s preventing”: a qualitative study of African female adolescent’s acceptability and preferences for proxy HIV prevention methods in Cape Town, South Africa
Source: BMC Public Health. 2023 Nov 2;23:2143. doi: 10.1186/s12889-023-16955-3 (PMC10621226; doi:10.1186/s12889-023-16955-3)
Supplement: Supplementary file 1 — Additional file 1. [file 12889_2023_16955_MOESM1_ESM.docx]

**CHAMPS FOCUS GROUP DISCUSSION GUIDE: UChoose**

**GROUP: 15 - 19 year old adolescent girls**

**Interviewer introduction:**

Thank you to everyone for joining us today. We are interested in your experiences and your opinions based on your use of contraception methods and hope that you will share your opinions in a safe, honest, and non-judgmental open conversation.  Each of you may be on two method or more methods and this is ok because we want to understand why you like or dislike the method you are/were on. This is a confidential meeting, meaning that your responses will remain anonymous, and that the members of this group should not discuss what is said with anyone else outside of the group. I will begin by asking some questions. Please remember that there are no right or wrong answers and your opinion counts.

| *Sample introduction of NuvaRing for local staff members.*    The birth control ring (AKA NuvaRing) is a safe, simple, and affordable birth control method that you wear inside your vagina. The small, flexible ring prevents pregnancy by releasing hormones into your body. The ring is really effective if you always use it the right way.  It’s easy to learn how to use NuvaRing. First, be sure the ring hasn’t expired (the expiration date will be on the package). Wash your hands with soap and water, then squeeze the sides of the ring together with your fingers and gently push it into your vagina. Don’t worry about the exact placement — if you can’t feel it when you’re walking around, it’s in right.  While NuvaRing is really good at preventing pregnancy, it won’t protect you from sexually transmitted infections. |
| --- |

**Content Areas and Questions:** (The questions to be asked are numbered, with further probes given beneath)

**EXPERIENCE OF USING STUDY PRODUCTS**

1. *Can you tell me about the contraception method you are using or have used in the past?*
2. *Which one did you like better? Why?* ***[Probe: dosing, route of admin, etc]***
3. *Which one did you like less? Why?* ***[Probe: dosing, route of admin, etc]***
4. *Can you tell me any difficulties you may be experiencing?*
5. *Which product do you think other girls in your community would prefer? Why?*

1. *What do you think of the dosing strategies of each product that you have tried? By this we mean route of administration as well as dosing regimen. Do you like / dislike the dosing? Why?*
2. *Pills / injection*
3. *Consider if you were to use the vaginal ring, you would have to change it once a month. Do you like / dislike the dosing? Why?*
4. *Pills / injection?*
5. *Vaginal ring?*

1. *What do you think are the benefits of each method?*
2. *Pills / injection?*
3. *Vaginal ring?*
4. *What do you think are the disadvantages of each method?*
5. *Pills / injection?*
6. *Vaginal ring?*

**ADHERENCE**

1. *How easy/difficult was it to take the products as you were instructed to?*
2. *Pills / injection?*
3. *Vaginal ring?*
4. *What are some of the challenges / barriers that you experienced which made it difficult for you to take / use each product as directed?*
   - 1. *Pills / injection?*
     2. *Vaginal ring?*
5. *How did you overcome those challenges / barriers? What strategies did you try to ensure that you take study product as directed?*
6. *Pills / injection?*
7. *Vaginal ring?*
8. *What are some of the things that made it easier / helped you to take the product as directed? What would you suggest that others should use / try in order to successfully take the product?*
9. *Pills / injection?*
10. *Vaginal ring?*

**MOTIVATIONS TO PARTICIPATE ON THE STUDY**

1. *If you had the option of joining a study which offered the ring as a contraception method, what would motivate you to join the study?*
   - - - *What motivated you to join the study?*
2. *Did the reimbursement money motivate you?*
3. *Did the health care that you were going to receive at the clinic motivate you?*
4. *Is there anything else that has not been mentioned?*

1. *If you had the option of joining a study which offered the ring as a contraception method, what would motivate you to remain in the study throughout the study period?*
2. *What motivated you to remain in the study throughout the study period?*
3. *Did the reimbursement money motivate you?*
4. *Did the health care that you were going to receive at the clinic motivate you?*
5. *Is there anything else that has not been mentioned?*

**RISK & RISK COMPENSATION**

1. *How at risk did you think you were of becoming pregnant?*
2. *How at risk did you think you were of becoming infected with HIV?*
3. *What made you perceive yourself to be at risk / not at risk?*
   - 1. *What sort of behaviours were you engaging in?*

**FUTURE OF PREVENTION**

1. *If HIV prevention products were available in pill/injectable and/or vaginal ring form, would you use either of them?*
2. *Why/why not?*
3. *Which would you prefer? Why/ why not?*
4. *Is there another HIV prevention method that you would prefer more? Eg condoms. Why/why not?*
5. *If HIV prevention products were available in pill/injectable and vaginal ring form, and you were going to use one, where would you like to access it from? Why?*

**Interviewer closing statement:**

We have talked about many things today. Before we finish, I wonder if you have any questions for me or if you have any additional comments about your experience or about the discussion today?
